# Coding Transcript-Derived Small Interfering RNAs: Their Biogenesis and Molecular Function in Arabidopsis

**DOI:** 10.3390/ijms27041701

**Published:** 2026-02-10

**Authors:** Xintong Xu, Nier Chen, Xinwen Qing, Xiaoli Peng, Xiangze Chen, Beixin Mo, Yongbing Ren

**Affiliations:** Guangdong Provincial Key Laboratory for Plant Epigenetics, College of Life Sciences and Oceanography, Shenzhen University, Shenzhen 518060, China; xxt19950625@163.com (X.X.); nierchen1128@163.com (N.C.); coratheone@163.com (X.Q.); pengxiaoli@szu.edu.cn (X.P.); chenxz@szu.edu.cn (X.C.)

**Keywords:** ct-siRNAs, RQC, EIN5/XRN4, SKI2, DCL2, DCL4, RDR6, SGS3

## Abstract

Coding transcripts-derived small interfering RNAs (ct-siRNAs) have emerged as a special class of endogenous siRNAs and have been implicated in the regulation of gene expression in plants, particularly under conditions where RNA metabolic pathways are perturbed. When the RNA quality control (RQC) system is impaired, the aberrant mRNA fragments were converted to double stranded forms by RNA-directed RNA polymerase 6 (RDR6) with the assistance of Suppressor of Gene Silencing 3 (SGS3) and subsequently processed by DICER-LIKE proteins into 21-nt and 22-nt ct-siRNAs. The accumulation of ct-siRNAs and the resulting suppression of their cognate genes are usually associated with altered plant growth and stress response. In this review, we summarize our current understanding of the ct-siRNAs, particularly their biogenesis under different RNA metabolic defective conditions. Comparative analysis of these genetic contexts indicates that ct-siRNAs act through translation inhibition and/or mRNA cleavage, with regulatory outcomes influenced by siRNA length and genetic background. We further summarize the biological consequence of ct-siRNA accumulation, which are frequently associated with impaired plant growth and stress adaptation. Finally, we discuss current controversies on ct-siRNAs research and highlight key unsolved questions for future investigation. Collectively, this review highlights ct-siRNAs as a link between impaired RNA metabolisms and post-transcriptional gene silencing, with context-dependent effects on plant growth and stress responses.

## 1. Introduction

Plant small RNAs (sRNAs) are typically 21–24 nucleotides (nt) in length, and mainly include microRNAs (miRNAs) and small interfering RNAs (siRNAs), both of which regulate diverse biological processes through target RNA cleavage and/or translational inhibition [1,2,3]. In Arabidopsis, four Dicer-like enzymes (DCL1–4) are responsible for sRNA production [4]. miRNAs are a well-characterized class of sRNAs that are mainly produced by DCL1 [5]. In contrast, siRNAs comprise a more diverse group with multiple biogenesis pathways and are mainly produced by DCL2, DCL3 and DCL4 [5]. siRNAs are derived from perfectly paired dsRNA precursors formed by RNA-dependent RNA polymerase (RDR) proteins [6]. *Arabidopsis* DCL4, DCL2, and DCL3 produce 21-nt, 22-nt, and 24-nt siRNAs respectively, which in turn trigger different mechanisms of gene silencing [7]. The 24-nt siRNAs produced by DCL3 are mainly involved in transcriptional silencing of transposon elements (TE) and repeat elements (RE) through the RNA-directed DNA methylation (RdDM) pathway [8]. DCL4 produces various 21-nt siRNAs, including the bulk of antiviral siRNAs and endogenous siRNAs in the RNA interference (RNAi) pathway [9,10]. DCL2-cleavaged 22-nt siRNAs in plants are involved in a transitive and systemic spread of siRNAs especially in antiviral defense, a process referred to as secondary RNAi [11].

The most extensively studied RNAi silencing mechanism involves the degradation of target mRNAs. In this process, ARGONAUTE (AGO) proteins recruit siRNAs to complementary target mRNAs [12]. In plants, this is often triggered by 21-nt siRNAs produced by DCL4 [10,13,14]. An antagonistic effect between DCL2 and DCL4 has been observed in RISC-related siRNA production. For example, during the production of viral siRNAs, DCL2 can substitute for DCL4 when DCL4 activity is impaired, implying functional redundancy between DCL2 and DCL4 [15]. In addition, massive 22-nt coding transcripts-derived siRNAs (ct-siRNAs) were produced in *dcl4* mutant but the siRNA levels returned to normal in *dcl2dcl4* double mutant [16,17], suggesting that DCL4 strongly competes with DCL2 in siRNA production.

Once successfully processed, both miRNAs and siRNAs are loaded into AGO proteins to form RISC. Thus, DCLs act as sRNA biogenesis enzymes, while AGOs serve as effectors that mediate sRNA function [18]. If there is a perfect sequence complementarity between sRNA and the target mRNAs, mRNA cleavage followed by degradation occurs, leading to reduced protein production [19]. However, if incomplete base-pairing exists between siRNA and its target mRNA, gene silencing could occur via ribosome-mediated translational inhibition, which also results in reduced protein production [20].

RNA quality control (RQC) is an integral part of eukaryotic gene expression, and relies primarily on exonuclease to eliminate aberrant transcripts [21,22]. RQC-mediated RNA degradation typically proceeds in two directions. The 5′-3′ RNA degradation is performed by three XRN exoribonucleases: XRN2, XRN3 and XRN4 (also known as EIN5) [23,24,25], whereas the 3′-5′ degradation is conducted by the exosome complex and its co-factors [26,27]. The SKI complex, composed of SKI2, SKI3, and SKI8, is required to recruit the exosome to mRNA targets [28,29,30]. Typically, XRN4 and SKI2 are representative enzymes in 5′-3′ and 3′-5′ decay pathways, respectively, and simultaneous dysfunction of XRN4 and SKI2 lead to severe growth abnormality [31]. When the RQC pathway is impaired, aberrant transcripts are subjected to the post-transcriptional gene silencing (PTGS) pathway, contributing to plant growth defects [16,17,31].

Nucleotidyltransferases (NTPs) constitute a class of enzymes that tether additional nucleotides to the 3′ ends of various RNAs in a non-templated manner [32]. HEN1 SUPPRESSOR1 (HESO1) and UTP: RNA uridylyltransferase (URT1) are two representative NTPs that have been extensively studied [32]. URT1 has been reported to uridylate mRNAs to thereby affect their stabilities [33,34], and HESO1 may also play a role in this process [34]. It has been proposed that impairment of NTP function may compromise mRNA degradation, which may result in the generation of aberrant ct-siRNAs.

In this review, we summarize the current understanding of ct-siRNAs, focusing on their biogenesis, molecular functions, biological functions, as well as current controversies on their biological functions and key questions for future research.

## 2. Generation of ct-siRNAs

**Figure 1:**
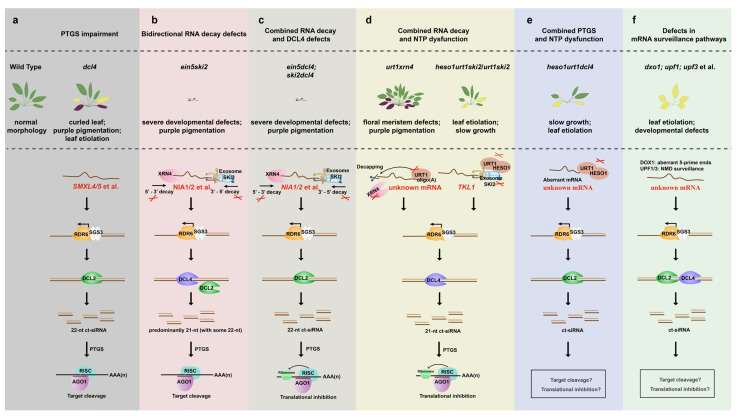
Overview of ct-siRNA biogenesis upon impairment of mRNA metabolic pathways and its roles in the repression of cognate genes. (**a**) Dysfunction of PTGS pathway as represented by *dcl4* mutant: massive 22-nt ct-siRNAs are generated from *SMXL4/5* loci and in turn repress the expression of *SMXL4/5* through mRNA cleavage. (**b**) Dysfunction of bidirectional mRNA decay pathways as represented by *ein5ski2* double mutant: massive 21- and 22-nt ct-siRNAs are generated from *NIA1/2* loci and in turn repress the expression of *NIA1/2* through mRNA cleavage. (**c**) Dysfunction of mRNA decay and PTGS pathways as represented by *ein5dcl4* and *ski2dcl4* mutants: massive 22nt ct-siRNAs are generated from *NIA1/2* loci and in turn repress the expression of *NIA1/2* through translational inhibition. (**d**) Dysfunction of NTP activity and mRNA decay pathway as represented by *urt1xrn4* and *heso1urt1ski2* mutants: *urt1xrn4* produces massive 21nt siRNAs from unidentified genes with unknown functions; whereas *heso1urt1ski2* produced massive 21-nt ct-siRNA from *TKL1* loci, which in turn suppresses the expression of *TKL1* through mRNA cleavage. (**e**) Dysfunction of NTP activity and PTGS pathway as represented by *heso1urt1dcl4* mutant: the ct-siRNAs and their cognate genes remain unidentified. (**f**) Dysfunction of key factors in mRNA surveillance pathway as represented by *dox1*, *upf1* and *upf3* mutants: the ct-siRNAs and their cognate genes remain unidentified. In (**a**–**f**), the impaired pathway is indicated by a scissor-like symbol.

DCL4 is a key factor in the PTGS pathway, and the *dcl4* mutant with defective phenotype produces massive 22-nt ct-siRNAs, which may affect plant phenotype through mRNA cleavage of cognate genes such as *SMXL4* and *SMXL5* (Figure 1a). EIN5 and SKI2 are two key factors in the mRNA decay pathway, which represent 5′-3′ and 3′-5′ decay respectively. The *ein5ski2* mutant displays severe growth arrest, and accumulates massive 22nt ct-siRNAs from gene loci like *NIA1* and *NIA2* (Figure 1b). When both mRNA decay and PTGS pathways are impaired, as exemplified by the *ein5dcl4* and *ski2dcl4* double mutants, massive 22nt ct-siRNAs are generated from gene loci like *NIA1* and *NIA2*, which in turn repress the expression of their cognate genes through translational inhibition (Figure 1c). NTPs can significantly influence the stability of mRNAs, and simultaneous inhibition of NTPs and RNA decay pathway contribute to the production of ct-siRNAs, which in turn regulate plant phenotype (Figure 1d). Simultaneous inhibition of NTPs and the PTGS pathway also results in ct-siRNAs production and affects plant phenotype. Finally, the dysfunction of some key factors in the mRNA surveillance pathway also causes ct-siRNAs generation and affects plant phenotype (Figure 1f).

### 2.1. Core Enzymes Involved in ct-siRNAs Generation

ct-siRNAs are typically generated when the RNA decay pathway is impaired, and its molecular mechanism is similar to the biogenesis of trans-activating siRNAs [9,34]. Undegraded single RNA fragments are first captured and converted into double-strand RNAs by the RNA-dependent RNA polymerases (RDRs), usually RDR6, with the assistance of SGS3. The dsRNAs are then processed to 21-nt and 22-nt ct-siRNAs by DCL4 and DCL2, respectively. The mature ct-siRNAs are then loaded to AGO1, where they suppress their cognate genes, through mRNA cleavage and/or translation inhibition. Thus, RDR6, SGS3, DCL2, DCL4 and AGO1 are the key regulators in ct-siRNAs production.

### 2.2. ct-siRNA Biogenesis upon Impairment of the PTGS Pathway

DCL4 is a key factor in PTGS pathway, and is mainly responsible for the production of various 21-nt siRNAs [35]. Phenotypic analysis showed that a certain proportion of *dcl4* mutants displayed aberrant morphological phenotypes, mainly including purple plants, yellow plants, purple and yellow plants, and dead plants [16,36]. Small RNA sequencing showed that the purple *dcl4* plants, while not the green *dcl4* plants, accumulated massive 22nt ct-siRNAs, which were mainly produced from gene loci *SMXL4* and *SMXL5* [16]. However, another study reported that apart from *SMXL4* and *SMXL5*, other gene loci such as *NIA1* and *NIA2* also showed the accumulation of abundant 22nt ct-siRNAs [36], though it remained unclear whether these aberrant ct-siRNAs were specifically produced in abnormal plants.

### 2.3. ct-siRNA Biogenesis Is Triggered by Defects in Bidirectional mRNA Decay Pathway

RNA surveillance systems are necessary for genome stability and proper gene expression, and are conserved across eukaryotes [37]. The mechanisms underlying both cytoplasmic and nuclear mRNA surveillance have been extensively summarized elsewhere [38,39]. In *Arabidopsis*, the simultaneous disruption of bidirectional cytoplasmic RNA decay pathways leads to pleiotropic plant developmental defects, accompanied by the production of massive ct-siRNAs from a subset of gene loci [31]. The *ein5ski2* double mutant in which the cytoplasmic RNA decay pathway is disrupted, displayed severe growth arrest, and a pronounced accumulation of mixed 21- and 22-nt ct-siRNAs. Two classes of gene loci—miRNA-targeted (e.g., *ARF6*, *REV* and *ATHB8*) and non-miRNA-targeted (e.g., *NIA1* and *NIA2*)—were identified as sources of these 21- and 22-nt ct-siRNAs. The arrested growth of *ein5ski2* was completely rescued in the *ein5ski2rdr6* triple mutant, and correspondingly, the ct-siRNAs were simultaneously restored to normal levels [31].

### 2.4. Synergistic Effects of mRNA Decay and PTGS on ct-siRNA Biogenesis

The PTGS pathway is a biological process in which gene expression is suppressed after transcription, and plays a crucial role in regulating gene expression, defending against viruses, and maintaining genomic stability in eukaryotes [38,39,40,41]. This process is initiated by the synthesis of dsRNAs, which are cleaved by the DCLs into siRNAs with different sizes [10,35,42,43]. It was observed that both *ein5dcl4* and *ski2dcl4* mutants displayed severely arrested growth, similar to that of *ein5ski2*, and that this phenotype was completely rescued in *ein5dcl4dcl2* and *ski2dcl4dcl2* mutants [17,31]. A similar rescue was also observed in the *ein5dcl4rdr6* and *ski2dcl4rdr6* [17]. Further analysis showed that 22nt ct-siRNAs were remarkably accumulated in *ein5dcl4* and *ski2dcl4* double mutants, and restored to normal levels in *ein5dcl4dcl2* and *ski2dcl4dcl2* triple mutants. A series of cognate genes that generate 22nt ct-siRNAs were identified in *ein5dcl4* and *ski2dcl4*. Among them, *NIA* and *NIA2*, two key genes involved in nitrogen transporting, were identified as the main contributors in the production of 22nt ct-siRNAs [17].

### 2.5. ct-siRNA Biogenesis Is Associated with NTP Dysfunction Under Compromised mRNA Decay or PTGS

HESO1 and URT1 are two representative NTPs in *Arabidopsis*, which modify RNAs and affect its stability. It was observed that the *urt1xrn4* displayed severely impaired statures and inflorescences development, and further analysis showed that *urt1xrn4* also accumulated massive 21nt ct-siRNAs [33]. The developmental defects of *urt1xrn4* were largely suppressed in the *urt1xrn4dcl2dcl4* quadruple mutant, which demonstrated the causality between *urt1xrn4* phenotype and 21nt ct-siRNAs production. Furthermore, the author also provided evidence that these 21nt ct-siRNAs were generated from aberrant mRNA intermediates resulting from impaired RNA decay.

In another study, the *heso1urt1ski2* triple mutant also displayed abnormal phenotype, which was rescued in the *heso1urt1ski2rdr6* quadruple mutant [44]. Similarly, the *hesolurt1dcl4* triple mutant also displayed abnormal phenotype, which was also rescued in the *hesolurt1dcl4dcl2rdr6* quadruple mutant. Further analysis showed that 21nt ct-siRNAs were significantly enriched in *heso1urt1ski2* triple mutant, which were restored to normal levels in *heso1urt1ski2rdr6* quadruple mutant. Importantly, *TKL1*, an essential gene involved in photosynthesis, accounted for a large portion of the 21nt ct-siRNAs in the *heso1urt1ski2* triple mutant, and the *tkl1* mutant also displayed phenotype similar to that of *heso1urt1ski2*. Furthermore, the *TKL1* expression in *heso1urt1ski2* was also significantly down-regulated [44]. Though it remains unclear whether HESO1 and URT1 tail the TKL1’s mRNA together and reduce its stability, these results provide clear evidence that 21nt ct-siRNAs directly target their cognate gene and reduce its expression.

### 2.6. ct-siRNA Accumulation Resulting from Defects in mRNA Surveillance

Eukaryotes possess three major types of mRNA surveillance pathways that prevent the accumulation of aberrant mRNAs, including nonsense-mediated decay (NMD), non-stop decay (NSD), and no-go decay (NGD) [45,46]. The NMD pathway degrades mRNAs with premature termination codons, the NSD pathway eliminates mRNAs lacking translation termination codons, and the NGD pathway targets mRNAs with sequences that contribute to ribosome stalling. Defects in the mRNA surveillance pathway may generate substrates for the RNAi machinery, which results in the production of aberrant ct-siRNAs [47]. *DXO* family proteins participate in mRNA cap surveillance, and the *dxo1* mutant displays a strong accumulation of 21-nt and 22-nt ct-siRNAs and severe growth defects [48], but it is unclear whether these phonotypes are directly caused by increased ct-siRNA levels. Similarly, UPF1 and UPF3 are two key factors of the NMD pathway, and the *upf1* and *upf3* mutants also accumulated massive ct-siRNAs [47]. Both *upf1* and *upf3* displayed growth defects [49], but it is also unclear whether the observed phenotypes are caused by ct-siRNA accumulation.

### 2.7. Features of Cognate Genes Prone to ct-siRNAs Generation

To date, over several hundred gene loci have been identified as sources of ct-siRNAs. For example, statistical analyses indicate that in the *ski2xrn4* double mutant, more than 441 genes produce ct-siRNAs [50]. Among these genes, some are miRNA targets, whereas others are not [31], indicating that ct-siRNA production is not strictly dependent on miRNA-mediated mRNA cleavage. A recent study has outlined several characteristics of these genes that selectively generate ct-siRNAs [50]. First, as source genes, their propensity to generate ct-siRNA is not associated with their cellular expression levels, but is instead correlated with their biological functions. Take *ein4dcl4* mutant as an example, genes producing relatively high levels of ct-siRNA are mainly involved in nitric oxide biosynthesis, nitrate assimilation, and response to light or hormone stimuli, whereas genes producing relatively lower levels of ct-siRNAs tend to regulate cell death, photosynthesis, auxin and hormone transport, and development. Second, these genes tend to have longer sequences and possess extended 5′ UTRs, whereas no clear correction is observed with 3′ UTRs or intron number. Finally, ct-siRNA-producing genes often exhibit higher GC content in their sequences [50]. Overall, the source genes that selectively generate ct-siRNAs are closely associated with their biological functions, sequence length, and GC content.

## 3. Molecular Functions of ct-siRNAs

### 3.1. ct-siRNA-Mediated Cognate mRNA Cleavage

In the purple *dcl4* mutant, massive 22-nt ct-siRNAs are mainly produced from the *SMXL4* and *SMXL5* loci, and the expression levels of *SMXL4* and *SMXL5* are significantly reduced [16]. By contrast, their expressions in the green *dcl4* remain unchanged. Given the low abundance of 21-nt ct-siRNAs in the purple *dcl4*, it was concluded that the 22-nt ct-siRNAs are loaded into AGO1 and mediate mRNA cleavage of their source genes [16]. Similarly, in the *ein5ski2* double mutant, the expression levels of source genes, including miRNA-targeted genes such as *ARF6/8*, *ATHB15* and *REV*, and non-miRNA-targeted genes such as *NIA1* and *NIA2*, are also significantly reduced [31]. This observation provides further supports for the role of ct-siRNAs in mediating cleavage of their cognate mRNAs. Notably, because both 21-nt and 22-nt ct-siRNAs accumulate in *ein5ski2*, it remains difficult to determine whether the decreased expression of source genes is attributable specifically to 21-nt ct-siRNAs, 22-nt ct-siRNAs, or both. Meanwhile, the expression of *TKL1* in the *heso1urt1ski2* triple mutant is also significantly reduced, and the corresponding TKL1 protein level is also remarkably decreased [44]. It is worth noting that, in comparison to 21-nt ct-siRNAs, 22nt ct-siRNAs are not noticeably increased in *heso1urt1ski2.* These observations indicate that the mRNAs of source genes are cleaved by the 21-nt ct-siRNAs in *heso1urt1ski2*. However, whether the 21-nt ct-siRNAs in *heso1urt1ski2* simultaneously mediate translational inhibition is still unclear.

### 3.2. ct-siRNA-Mediated Translational Inhibition

In the *ein5dcl4* and *ski2dcl4* mutants, massive accumulation of 22-nt ct-siRNAs is observed, whereas the expression levels of their source genes, such as *NIA1*, *NIA2*, *SMXL4* and *SMXL5*, are irregularly changed [31]. By contrast, the protein levels of these genes are dramatically and consistently reduced. Polysome profiles show that the overall translation activities in *ein5dcl4* and *ski2dcl4* are significantly decreased, with particularly strong repression observed for source genes such as *NIA1* and *NIA2*. In an artificially assembled RISC system, synthesized 22-nt ct-siRNAs tend to repress target protein synthesis more efficiently than 21-nt ct-siRNAs, further supporting a role for 22-nt ct-siRNAs in translational inhibition [31]. Due to the dysfunction of DCL4, the *ein5dcl4* and *ski2dcl4* specifically generates 22nt ct-siRNAs but not 21-nt ct-siRNAs. Together, these observations provide strong evidence that 22-nt ct-siRNAs specifically mediates translation inhibition in these genetic backgrounds.

## 4. Biological Functions of ct-siRNAs

### 4.1. Roles of ct-siRNA in Plant Growth Regulation

In one study, two types of *dcl4* mutants exhibiting distinct phenotypes were separately analyzed, namely the purple *dcl4* mutant and the green *dcl4* mutant [16]. The purple *dcl4* mutant displayed purple leaves and accumulated massive 22-nt ct-siRNAs, while the green *dcl4* mutant displayed normal leaves and contained normal 22-nt ct-siRNAs. However, the shoot size of the purple *dcl4* mutant was not significantly reduced. The purple *dcl4* phenotype was fully rescued in *dcl4dcl2*, *dcl4rdr6* and *dcl4sgs3* double mutants. Accordingly, the increased levels of 22-nt ct-siRNAs observed in the purple *dcl4* mutant were restored to normal levels in the *dcl4dcl2* and *dcl4rdr6* double mutants. Based on these observations, accumulation of 22-nt ct-siRNAs appear to be positively correlated with the phenotypic manifestation of the *dcl4* mutant. In another study, abnormal phenotypes were also observed in the *dcl4* mutant plants [51]. The shoot phenotypes of *dcl4* plants were mainly divided into four types, the normal, the yellow, the purple (named anthocyanin in the text), and the purple plus yellow. It seems that the shoot size of the abnormal *dcl4* mutants was moderately reduced compared with that of the wild-type plants. The aberrant phenotype of *dcl4* was also rescued in the *dcl4 dcl2* and *dcl4 rdr6*s. Accordingly, the 22-nt ct-siRNAs in *dcl4* were also restored to normal levels in *dcl4 dcl2*. Interestingly, the incompletely penetrated phenotype of *dcl4* became fully penetrated in *dcl4 sgt1b*, which was also rescued in *dcl4 sgt1b dcl2* [52].

The *ein5ski2* mutant plants displayed severe growth defects, including significantly decreased shoot size and purple leaves, which were rescued in *ein5 ski2 rdr6*, *ein5 ski2 dcl2* and *ein 5ski2 dcl4 dcl2* mutants [31]. The vastly accumulated 21-nt and 22-nt ct-siRNAs in *ein5 ski2* were also restored to normal levels in *ein 5ski2 rdr6*. These results indicate that the mixed 21-nt and 22-nt ct-siRNAs in *ein5ski2* are also a positive correlation between ct-siRNA accumulation and growth defects in *ein5 ski2*. Meanwhile, the *ein5 dcl4* and *ski2 dcl4* plants also displayed phenotypes similar to those of *ein5ski2*, and these phenotypes were also completely rescued in the corresponding *rdr6* and *dcl2* mutant backgrounds, such as in *ein5dcl4rdr6* and *ski2dcl4rdr6*, and *ein5dcl4dcl2* and *ski2dcl4dcl2* mutants [17]. Consistently, the largely increased 22-nt ct-siRNAs in *ein5dcl4* and *ski2dcl4* were also restored to normal levels in *ein5dcl4rdr6* and *ski2dcl4rdr6*, and *ein5dcl4dcl2* and *ski2dcl4dcl2* mutants. These findings further validated a negative role for 22-nt ct-siRNAs in plant growth regulation in *ein5dcl4* and *ski2dcl4* mutant plants. In addition, the defective phenotypes of *ein5dcl4* and *ski2dcl4* were also completely rescued in *ein5dcl4ago1* and *ski2dcl4ago1* mutants, and partially rescued in *ein5dcl4hen1* and *ski2dcl4hen1* [17]. These observations further suggested that inhibition of the PTGS pathway could alleviate the detrimental effects of 22-nt ct-siRNAs on plant growth.

The *urt1xrn4* mutant plant also displayed severely impaired statures and failed to develop inflorescences, and these defects were rescued in *urt1xrn4dcl2dcl4*, which validated the causality between *urt1xrn4* phenotype and the production of ct-siRNAs [33]. However, although the *urt1xrn4* mutant also displayed purple leaves, its shoot size was not significantly altered. In another study using the Landsberg ecotype, the *heso1urt1ski2* mutant also displayed abnormal phenotypes, particularly yellow leaves, which were also observed in the *heso1urt1dcl4* mutant; their phenotypes were rescued in *heso1urt1ski2* and *heso1urt1ski2dcl4rdr6*, respectively [44]. It was demonstrated that the abnormal phenotype of *heso1urt1ski2* was caused by the increased 21-nt ct-siRNAs from *TKL1*, a photosynthesis-related gene [44]. In contrast, a separate study using the Columbia ecotype, reported that *ski2urt1* and *rrp4urt1,* but not *ski2heso1* and *rrp4heso1,* displayed abnormal phenotypes, including small shoots, and purple and yellow leaves [32]. RRP4, a core subunit of exosome, was expected to act in a similar role as SKI2 in the RQC pathway. Expectedly, the aberrant phenotype of *rrp4urt1* was rescued in *rrp4urt1dcl2* and *rrp4urt1rdr6*. Meanwhile, the phenotype of *rrp4urt1* was surprisingly rescued in *rrp4urt1heso1*, though the yellow leaves of *ski2urt1heso1* were still observed [32]. However, shoot morphology data and ct-siRNA levels were not reported for *ski2urt1heso1*. It is still unclear whether the phenotype difference in *ski2urt1heso1* between these two studies was caused by different ecotype backgrounds.

Dysfunction of several key factors in mRNA surveillance pathways, such as *DXO1, UPF1* and *UPF3* also leads to the production of massive ct-siRNAs, and mutants of these genes also displayed severe growth defects, such as small shoots and yellow leaves [48]. However, the causality between ct-siRNAs and plant phenotypes in these mutants are still unclear.

### 4.2. Roles of ct-siRNA in Stress Adaptation

To date, two studies have clearly revealed both positive and negative roles of ct-siRNAs in plant response to abiotic stresses. In one study, the total amount of ct-siRNAs was noticeably induced even in wildtype plants under nitrogen deficient conditions, with a more obvious increase observed in the *dcl4* mutant. Consistently, *dcl4* plants displayed hypersensitive phenotypes when grown under nitrogen deficient conditions, such as purple shoots and short roots [31]. In another study, the *ski2xrn4* seedlings displayed strong growth arrest, but exhibited enhanced thermotolerance, as indicated by a much higher survival rate and greater relative fresh weight compared to wildtype seedlings under heat stress [53,54]. These observations suggested that, although ct-siRNAs can be deleterious to plant growth, they may confer beneficial effects on stress resistance under certain conditions. Thus, the actual roles (positive or negative) of ct-siRNAs in stress adaptation appear to depend on the biological roles of their source genes.

## 5. Current Controversy Regarding the Role of ct-siRNAs in Plant Growth

The aberrant phenotypes observed in *dcl4*, *ein5ski2*, *ein5dcl4*, *ski2dcl4*, *heso1urt1ski2* and *heso1urt1dcl4*, accompanied by the dramatically increased ct-siRNAs in these genotypes, provide persuasive evidence that ct-siRNAs act as negative modulators of plant growth. However, a recent study has provided an alternative perspective on this issue [51,55]. In that study, reducing the dosage of DCL2 protein in *dcl4dcl2/+* (with *DCL2* in heterozygous state) had little effect on ct-siRNAs abundance, but the plant growth phenotype was astonishingly rescued, which indicated an indirect correction between ct-siRNA levels and plant phenotype. This result was simultaneously observed using two distinct *dcl2* alleles. Furthermore, a similar result was also observed using *dcl4sgt1b* with a complete penetrated phenotype.

These two perspectives have been summarized in two recently published articles [56,57]. Both viewpoints are supported by a wealth of experimental data and appear well grounded, which makes readers particularly interested in research in this field. Controversies in scientific research are quite common and often stimulate further investigation. In this context, continued studies employing additional genetic backgrounds and experimental conditions will be essential to clarify the precise roles of ct-siRNAs in plant growth regulation.

## 6. Conclusions and Future Perspectives

The RQC system is vital for plant growth and development. When the RQC system is impaired, aberrant transcripts are redirected to the PTGS pathway, and processed into massive aberrant siRNAs. When these siRNAs are produced from coding transcripts, they are named as ct-siRNAs. These ct-siRNAs subsequently bind to their cognate transcripts and impair gene functions through mRNA cleavage and/or translation inhibition. Due to the weakened function of their source genes, ct-siRNAs are generally associated with a negative effect on plant growth. However, under certain conditions, these seemingly deleterious ct-siRNAs may be beneficial for stress adaption.

Although significant progress has been made in ct-siRNAs research, particularly regarding the mechanism of their biogenesis, several important questions remain to be addressed. First, additional genetic materials that produce ct-siRNAs in large quantities need to be identified, particularly those that generate ct-siRNAs of varying lengths from different cognate genes under distinct conditions. Second, the correlation between ct-siRNAs generation and environmental stimuli should be elucidated. Third, since the currently observed ct-siRNAs are primarily produced in various mutants, their production in wild-type plants under specific conditions warrants special investigation. Fourth, whether ct-siRNAs are broadly involved in the regulation of stress adaptation needs to be clarified. Fifth, it remains to be determined whether certain types of ct-siRNAs promote plant growth. Finally, the direct or indirect causal relationship between ct-siRNA accumulation and plant phenotypes should be explicitly established.

## Data Availability

We confirm that all data used in the article are obtained from published articles and their supplementary materials.

## Abbreviations

The following abbreviations are used in this manuscript:

AGO1	ARGONAUTE 1
ct-siRNAs	Coding transcripts-derived small interfering RNAs
DCL	DICER-LIKE
dsRNAs	Double strand RNAs
HESO1	HEN1 SUPPRESSOR1
nt	Nucleotide
NGD	No-go decay
NMD	Nonsense-mediated decay
NSD	Non-stop decay
NTPs	Nucleotidyltransferases
RdDM	RNA-directed DNA methylation
RDR6	RNA-directed RNA polymerase 6
RE	Repeat elements
RISC	RNA-induced silencing complex
RNAi	RNA interference
RQC	RNA quality control
SGS3	Suppressor of Gene Silencing 3
sRNAs	Small RNAs
TE	Transposon elements
URT1	UTP: RNA uridylyltransferas
